# Obesity, diet, and inflammation in Filipino schoolchildren: an opinion paper

**DOI:** 10.3389/fnut.2026.1896799

**Published:** 2026-08-14

**Authors:** Vheejay B. Tampol

**Affiliations:** Department of Biochemistry, Molecular Biology and Nutrition, Faculty of Medicine and Surgery, University of Santo Tomas, Manila, Philippines

**Keywords:** Filipino schoolchildren, inflammation, micronutrient status, NOVA classification, obesity, ultra-processed foods

## 1 Introduction

The global surge in childhood obesity, concurrent with persistent micronutrient deficiencies, presents a profound nutritional paradox that disproportionately burdens low- and middle-income countries (LMICs). Two recent investigations by Amarra et al. (1, 2) provide critical epidemiological insights into this dual burden among a cohort of 166 apparently healthy Filipino schoolchildren aged 5 to 9 years in Metro Manila. The initial research article entitled “Obesity and micronutrient status predict inflammation and weaker immune function in Filipino schoolchildren” published in February 2025 reveals a high prevalence of Epstein-Barr virus (EBV) seropositivity and demonstrates that overweight/obesity, low serum vitamin C, and elevated iron levels are significantly associated with CRP-defined inflammation and abnormal neutrophil profiles. Their subsequent brief research report entitled “Processed snacks and baked goods predict inflammation risk in Filipino schoolchildren” published in May 2025 shifts the focus to dietary patterns, demonstrating that the habitual consumption of ultra-processed foods (UPFs), specifically commercial baked goods, cereals, and processed rice snacks, is significantly correlated with elevated inflammatory biomarkers.

To delineate the dietary classification framework utilized in this analysis and to be consistent with the classification used in the second study of Amarra et al. (1) this paper adopts the internationally recognized NOVA food classification system, which categorizes food products into four distinct groups based on the extent and purpose of industrial processing, independent of their nutrient profile (3). While “processed foods” (NOVA Group 3, such as canned vegetables, salted nuts, or artisanal cheeses) undergo basic preservation methods to extend shelf life, “ultra-processed foods” (UPFs; NOVA Group 4) are industrial formulations containing minimal intact whole foods. These products are characteristically formulated with additives, such as emulsifiers, stabilizers, and colorants, designed to induce hyper-palatability. Although the second study of Amarra et al. (2) occasionally utilizes the terms “processed snacks,” “processed foods,” and “ultra-processed foods” interchangeably, the specific dietary items highlighted in their cohort, including commercial baked goods, instant noodles, and packaged cereals, fall unequivocally under the classification of ultra-processed foods (NOVA Group 4).

Amarra et al. (1, 2) were able to elucidate the epidemiological associations among diet, anthropometry, and systemic inflammation, but their analyses on the underlying mechanisms can be further expanded. A comprehensive interpretation of these findings necessitates a multifaceted approach that extends beyond isolated nutritional metrics. To this end, the specific contribution of this opinion paper is threefold:

first, it contextualizes the findings of Amarra et al. (1, 2) within the broader socioeconomic reality and the rapid nutrition transition of the Philippines; second, it critically evaluates the methodological and analytical limitations of the cross-sectional study designs; and third, it proposes the gut microbiome-immunity axis as a novel conceptual framework to direct future longitudinal and mechanistic studies on pediatric metabolic inflammation.

## 2 Discussion

### 2.1 Interpreting findings in the Philippine context

An accurate interpretation of these results must be contextualized within the framework of the double burden of malnutrition, a phenomenon wherein adiposity and micronutrient deficiencies co-occur at the population, household, and individual levels. The Philippines is currently undergoing a rapid and multifactorial nutrition transition, rendering the population highly susceptible to this dual burden (4). An escalating proportion of Filipino youth and adults exhibit both obesity and critical micronutrient inadequacies, a paradigm shift driven predominantly by sweeping structural and macroeconomic changes (5).

Historically, pediatric malnutrition in the Philippines was characterized predominantly by stunting and wasting. Contemporary epidemiological trends, however, indicate a significant shift. Obesogenic environments are proliferating, concomitantly driving the incidence of childhood obesity (6). Rapid urbanization and pervasive food marketing have facilitated the displacement of traditional, nutrient-dense diets by highly caloric, nutrient-depleted UPFs. This dietary transition is increasingly evident even in rural locales, where adolescent consumption of nutrient-dense whole foods remains alarmingly deficient (7).

The high frequency of instant noodle, packaged snack, and commercial baked good consumption reported by Amarra et al. (2) cannot be attributed exclusively to individual behavioral decisions. Rather, these consumption patterns are indicative of systemic food insecurity and an increasingly industrialized food environment, which collectively predispose Filipino youth to early-onset metabolic dysfunction and immunological vulnerability.

### 2.2 Methodological critique of the studies

A notable methodological strength of the studies by Amarra et al. (1, 2) lies in their focus on public schoolchildren in the Philippines, a highly vulnerable and frequently underrepresented demographic in global nutritional literature. By adjusting for key covariates such as maternal education, sex, and body mass index (BMI), the researchers provided a robust epidemiological foundation linking early dietary exposures to immune health (8).

As is typical of preliminary studies, the relatively modest sample size of schoolchildren (*N* = 166) and the restriction to a localized cohort within Metro Manila limits the generalizability of the findings across the diverse pediatric populations of the Philippines, particularly those residing in rural or marginalized regions.

Looking at the dietary assessment method, the use of two non-consecutive 24-h dietary recalls administered by trained interviewers to the schoolchildren's mother or caretaker captured the variation in eating patterns. But the reliance on proxy informants such as parents or caretakers inherently introduces recall bias. Episodic dietary assessments, such as 24-h dietary recalls, are often insufficient to capture the chronic, long-term exposure to UPFs that is physiologically requisite for the pathogenesis of low-grade systemic inflammation (9).

A qualitative, binary C-reactive protein (CRP) assay was used to assess for the presence of inflammation among the schoolchildren. While a qualitative assay is cost-effective and easily screens for inflammation, it does not allow for the identification of dose-response relationships between dietary intake and inflammatory severity. Low-grade systemic inflammation, frequently mediated by adiposity and UPF consumption, is typically characterized by subclinical elevations in inflammatory cytokines. A qualitative assay lacks the analytical sensitivity necessary to detect these physiological nuances, which are optimally quantified via high-sensitivity assays (hs-CRP) (10).

Moreover, baseline inflammatory markers and leukocyte profiles in pediatric cohorts are inherently dynamic and can be modulated by unmeasured variables, including, subclinical or recent acute infections, the state of biological maturation, physical activity levels, and social determinants of health. Parasitic and viral infections are endemic in Philippine public school settings and can independently elevate acute-phase reactants, alter neutrophil kinetics, and disrupt iron homeostasis (11, 12). As for biological maturation, it profoundly influences body composition, metabolic signaling pathways, and basal inflammatory states (13, 14). Regular physical activity of at least moderate intensity is a potent independent modulator of not only physical health, but also of metabolic and mental health, and play a crucial role in the treatment of children with chronic inflammatory diseases such as obesity and juvenile idiopathic arthritis (15). Social determinants of health such as the socioeconomic and the household food security statuses of the schoolchildren's family also shape their dietary patterns (16) and contribute to their allostatic load (17, 18).

Finally, the inherent limitation of a cross-sectional research design precludes the establishment of causal directionality. It remains undetermined whether micronutrient inadequacies and UPF consumption precede the onset of inflammation, or whether these associations are confounded by reverse causality and undiagnosed physiological stressors.

### 2.3 Assessment of scientific hypotheses and mechanistic pathways

#### 2.3.1 Vitamin C and immune function

The initial study of Amarra et al. (1) suggests that suboptimal vitamin C status is associated with heightened inflammatory markers, a hypothesis robustly supported by contemporary immunological literature. Vitamin C functions as a critical antioxidant, protecting immune cells from endogenous reactive oxygen species (ROS) generated during pathogen eradication. Furthermore, it is essential for the maintenance of epithelial barrier integrity, leukocyte chemotaxis, and phagocytic efficacy (19, 20).

Additionally, vitamin C exerts significant epigenetic influence, serving as a required cofactor for enzymes regulating T-cell differentiation (21). Consequently, in states of vitamin C deficiency, host cellular immune surveillance may become compromised, plausibly facilitating the subclinical reactivation of latent viral pathogens such as EBV (22, 23).

#### 2.3.2 Epstein-Barr Virus (EBV) exposure

The reported high prevalence of EBV must be viewed in the following aspects: (first) its role as an indicator of environmental exposure, (second) a potential active immunomodulatory contributor, or (third) a substantive confounding variable within pediatric nutritional epidemiology.

- EBV as a Marker of Socio-Environmental and Cellular Immune Status: in low-resource and rapidly urbanizing settings, primary EBV infection typically occurs in early childhood, significantly predating the acquisition curves observed in high-income nations (24). This accelerated viral transmission is frequently facilitated by high-density housing and shared domestic environments. Consequently, elevated EBV seroprevalence functions as a structural proxy for socioeconomic vulnerability. Furthermore, maintaining latent herpesviruses requires constant metabolic and cellular expenditure by the host immune system; thus, elevated EBV antibody titers frequently serve as a sensitive biomarker of diminished cell-mediated immunity, reflecting subclinical, stress-induced, or nutritionally mediated declines in T-cell surveillance (25, 26).
- EBV as an Inflammatory Modulator: EBV is not immunologically inert during latency. When host nutritional status is compromised, particularly concerning deficiencies in T-cell modulators like vitamin C, immune surveillance may falter, permitting periodic, subclinical lytic reactivation of the virus (21). Active viral replication within B-lymphocytes and the mucosal epithelium can function as an endogenous inflammatory stimulus, triggering the release of pro-inflammatory cytokines such as interleukin-6 (IL-6), tumor necrosis factor-alpha (TNF-alpha), and interferon-gamma (IFN-gamma) (27). Thus, subclinical EBV reactivation may act synergistically with adiposity to perpetuate a systemic low-grade inflammatory state.
- EBV as a biologically confounding variable: clinical evidence demonstrates that active or reactivated EBV infection correlates with structural alterations in baseline leukocyte architecture and acute-phase reactants. EBV replication frequently induces transient atypical lymphocytosis, altered neutrophil-to-lymphocyte ratios, and elevated CRP concentrations (28). Given the cross-sectional design utilized by (1) it is difficult to determine whether nutritional inadequacies incited the inflammatory response or whether unmeasured subclinical EBV reactivation cycles independently modulated the elevated CRP and aberrant neutrophil counts. In the absence of quantitative viral load assays such as PCR or phase-specific serology to distinguish latent from reactivated states, EBV remains a significant unmeasured confounder.

#### 2.3.3 Iron metabolism and inflammation

Amarra et al. (1) also noted that elevated serum iron levels correlated with abnormal neutrophil profiles, suggesting an association between iron status and a pro-inflammatory milieu. Although the intersection of iron biology and immunology is complex, utilizing isolated elevations in serum iron as a definitive surrogate for clinical iron overload, tissue accumulation, or oxidative toxicity may lead to inaccuracies. Accurately characterizing iron-associated metabolic inflammation requires differentiation among distinct physiological compartments: serum iron, serum ferritin, transferrin saturation (TSAT), hepcidin, and non-transferrin-bound iron (NTBI).

- Serum iron vs. intracellular iron stores: serum iron quantifies the transient concentration of ferric iron (Fe^3+^) bound to circulating transferrin. This represents a highly labile pool subject to profound diurnal variation, acute dietary fluctuations, and physiological stress. It does not correlate reliably with intracellular iron reserves, which are typically indexed via serum ferritin. However, the interpretation of ferritin is subject to significant confounding in inflammatory states, as it functions as a classical acute-phase reactant. Elevated pro-inflammatory cytokines, notably IL-6, upregulate hepatic ferritin synthesis independent of total body iron stores (29). Consequently, hyperferritinemia in this cohort may solely reflect systemic inflammation rather than iron sufficiency.
- The Protective Role of Transferrin Saturation (TSAT) and Hepcidin: under homeostatic conditions, circulating iron is highly sequestered by transferrin, neutralizing its potential for redox toxicity. Transferrin saturation (TSAT) designates the proportion of transferrin occupied by iron, typically maintained between 20 and 45%. Systemic iron homeostasis is meticulously regulated by hepcidin, an acute-phase peptide hormone. Elevated circulating IL-6 potently stimulates hepcidin transcription, which subsequently induces the degradation of ferroportin, the primary cellular iron exporter. This cascade effectively arrests intestinal iron absorption and sequesters iron within macrophages, a host-defense mechanism termed nutritional immunity (30). Crucially, this sequestration mechanism typically reduces circulating serum iron levels. Therefore, the observation of elevated serum iron predicting inflammatory markers is biochemically paradoxical and strongly implies the presence of transient dietary variations or unmeasured pre-analytical confounding rather than pathological iron overload.
- The Threshold for Non-Transferrin-Bound Iron (NTBI) and Oxidative Toxicity: pathological iron-mediated oxidative toxicity is hypothesized to manifest only when the binding capacity of transferrin is overwhelmed, generally at a TSAT exceeding 70 to 80% (31). Under such conditions, a reactive fraction known as non-transferrin-bound iron (NTBI), encompassing labile plasma iron (LPI), emerges. These fractions participate in Fenton chemistry, catalyzing the generation of highly toxic hydroxyl radicals that induce lipid peroxidation, cellular apoptosis, and systemic inflammation (32). Notably, NTBI is physiologically absent at normal or moderately elevated serum iron levels when transferrin retains binding capacity.

Thus, attributing subclinical inflammation or aberrant neutrophil indices to oxidative iron toxicity or NTBI accumulation within this cohort remains highly speculative. Robust clinical conclusions regarding iron status in pediatric populations mandate the utilization of comprehensive panels inclusive of ferritin, TIBC to calculate TSAT, and hepcidin to prevent the mischaracterization of nutritional status and ensure the safety of public health supplementation initiatives.

#### 2.3.4 The ultra-processed food-gut microbiome hypothesis

The second study of Amarra et al. (2) associates UPF consumption with inflammation, attributing this observation predominantly to macronutrient profiles characterized by high simple sugars and saturated fats. Although high glycemic loads and trans fats undeniably contribute to a pro-inflammatory state, recent translational literature supports exploring a more intricate mechanistic hypothesis. It is proposed that the initial physiological insult driven by UPFs may manifest at the intestinal mucosa, mediated by the gut microbiome-immune axis (33, 34). It is imperative, however, to contextualize this pathway as a conceptual, theoretical model rather than an empirically validated mechanism within this specific demographic.

Preclinical data suggest that dietary emulsifiers such as carboxymethylcellulose, polysorbate 80, and synthetic lecithins, frequently incorporated into commercial baked goods and ultra-processed snacks to augment texture and extend shelf life, may compromise mucosal integrity (35, 36). It should be noted that the utilization, concentration, and formulation of these emulsifiers display marked heterogeneity depending on brand formulations and regional regulatory frameworks.

In highly controlled animal and *in vitro* models, exposure to these synthetic compounds alters the taxonomic composition of the gut microbiota and degrades the protective colonic mucus layer (37). This structural degradation facilitates microbiota encroachment, a phenomenon wherein luminal bacteria migrate and establish direct physical contact with the intestinal epithelium (38).

Theoretically, compromised mucosal integrity may precipitate increased intestinal permeability, allowing the translocation of highly immunogenic bacterial components, such as lipopolysaccharides (LPS), into the systemic circulation (39). Upon infiltrating the lamina propria, these elements trigger pattern recognition receptors on host innate immune cells, initiating a cascade of pro-inflammatory cytokines that eventually stimulate the hepatic acute-phase response and CRP synthesis (40).

While this gut-barrier-immune hypothesis is biochemically highly coherent, extrapolating these mechanisms from murine models to pediatric human cohorts necessitates profound caution. The baseline microbiomes of schoolchildren in LMICs are heavily modulated by distinct socio-environmental determinants, including endemic environmental enteric dysfunction (EED) and chronic exposure to geohelminths. These preexisting variables may fundamentally alter host-microbiome interactions in response to industrial food additives.

Given the absence of metagenomic sequencing or objective clinical biomarkers of gut barrier integrity, such as serum zonulin, intestinal fatty acid-binding protein, or lactulose-mannitol ratios, in the studies of Amarra et al. (2) this microbiome-mediated etiology remains an unvalidated, albeit highly compelling, hypothesis for future investigation.

## 3 Conclusion

In conclusion, the investigations by Amarra et al. (1, 2) furnish valuable epidemiological insights into the nutritional paradigm of Filipino schoolchildren, elucidating the troubling co-occurrence of adiposity and micronutrient deficiencies. Their foundational hypothesis that suboptimal micronutrient status and frequent ultra-processed food consumption are statistically associated with elevated systemic inflammation is biologically plausible and corroborated by broader international literature.

To advance beyond cross-sectional associations, future pediatric nutritional research in transitioning economies like the Philippines must be designed to integrate the following:

- Longitudinal cohorts: to monitor the ontogeny of low-grade systemic inflammation and map causal trajectories.
- Robust dietary assessment: employing validated, high-resolution food frequency questionnaires (FFQs) or multi-day dietary records to precisely quantify chronic exposure to ultra-processed foods (NOVA Group 4).
- Sensitive, multi-marker biological panels: transitioning to quantitative high-sensitivity CRP (hs-CRP) assays, concurrently measured with comprehensive iron profiles (ferritin, TSAT, and hepcidin) to rigorously control for acute-phase confounding.
- Intestinal barrier and microbiome profiling: utilizing metagenomic sequencing paired with objective serum biomarkers of mucosal integrity to empirically evaluate the proposed UPF-gut-barrier-immune hypothesis.

By adopting these integrated, multidisciplinary methodologies, subsequent research can systematically untangle the complex pathophysiological networks connecting modern industrialized food environments to chronic pediatric inflammation, thereby providing a more precise foundation for evidence-based public health interventions.
